# Bilateral multiple pulmonary artery aneurysms associated with cavitary pulmonary tuberculosis: a case report

**DOI:** 10.1186/s13256-017-1360-x

**Published:** 2017-07-19

**Authors:** Pedro Pallangyo, Frederick Lyimo, Smita Bhalia, Hilda Makungu, Bashir Nyangasa, Flora Lwakatare, Pal Suranyi, Mohamed Janabi

**Affiliations:** 1 Department of Cardiovascular Medicine, Jakaya Kikwete Cardiac Institute, P.O Box 65141, Dar es Salaam, Tanzania; 2 Department of Radiology, Muhimbili National Hospital, P.O Box 65000, Dar es Salaam, Tanzania; 3 Department of Cardiovascular Surgery, Jakaya Kikwete Cardiac Institute, P.O Box 65141, Dar es Salaam, Tanzania; 4 Department of Radiology and Radiological Science, Medical University of South Carolina, 25 Courteney Drive, MSC 226, Charleston, SC 29425 USA

**Keywords:** Pulmonary artery aneurysm, Pulmonary hypertension, PA aneurysm, PAA, tuberculosis, TB

## Abstract

**Background:**

Pulmonary artery aneurysms constitute <1% of aneurysms occurring in the thoracic cavity. Congenital cardiac defects are responsible for the majority (>50%) of cases, however, pulmonary artery aneurysm is a rare sequelae of pulmonary tuberculosis reported in about 5% of patients with chronic cavitary tuberculosis on autopsy. The natural history of this potentially fatal condition remains poorly understood and guidelines for optimal management are controversial.

**Case presentation:**

A 24-year-old man, a nursing student of African descent, was referred to us from an up-country regional hospital with a 4-week history of recurrent episodes of breathlessness, awareness of heartbeats and coughing blood 3 weeks after completing a 6-month course of anti-tuberculosis drugs. A physical examination revealed conjuctival and palmar pallor but there were no stigmata of connective tissue disorders, systemic vasculitides or congenital heart disease. An examination of the cardiovascular system revealed accentuated second heart sound (S_2_) with early diastolic (grade 1/6) and holosystolic (grade 2/6) murmurs at the pulmonic and tricuspid areas respectively. Blood tests showed iron deficiency anemia, prolonged bleeding time, and mild hyponatremia. A chest radiograph revealed bilateral ovoid-shaped perihilar opacities while a computed tomography scan showed bilateral multiple pulmonary artery pseudoaneurysms with surrounding hematoma together with adjacent cystic changes, consolidations, and tree-in-bud appearance. Our patient refused to undergo surgery and died of aneurismal rupture after 9 days of hospitalization.

**Conclusions:**

The presence of intractable hemoptysis among patients with tuberculosis even after completion of anti-tuberculosis course should raise an index of suspicion for pulmonary artery aneurysm. Furthermore, despite of its rarity, early recognition and timely surgical intervention of pulmonary artery aneurysm is crucial to reducing morbidity and preventing the attributed mortality.

## Background

Focal dilatation (>4 cm) [1–3] of the pulmonary arterial system is referred to as pulmonary artery aneurysm (PAA). Owing to its asymptomatic course in the majority of cases, this rare entity used to be an autopsy finding with prevalence rates ranging between 0.001% and 0.007% [4–7]. With the advent of a two-dimensional (2D)-echocardiography (ECHO), computed tomography (CT) scan and magnetic resonance imaging (MRI), more cases are now diagnosed often incidentally among living patients [8–13]. Nonetheless, the natural history of this potentially fatal condition remains poorly understood and guidelines for optimal management are controversial [3, 14–18].

Causes of PAA are numerous and diverse in pathogenesis, however, congenital cardiac defects (patent ductus arteriosus, ventricular and atrial septal defects) are implicated in about 50% of cases [19, 20]. Other causes include infections (tuberculosis, syphilis, mycotic aneurysms), systemic vasculitides (Behcet’s disease, giant cell arteritis), connective tissue disorders (Marfan’s syndrome, Hughes-Stovin syndrome), degenerative diseases (atherosclerosis), chest trauma, and idiopathic PAA [3, 4, 8, 9, 14, 16–24]. The clinical manifestations of PAA are largely nonspecific but dyspnea, palpitations, chest pain, cough, and hemoptysis are frequently reported in symptomatic patients [1–28]. Radiological imaging is essential in establishing the diagnosis as the nonspecific clinical findings are inevitably inconclusive. We report a case of bilateral multiple pulmonary artery aneurysms in a 24-year-old male nursing student from Tanzania.

## Case presentation

A 24-year-old man, a nursing student of African descent, was referred to us from an up-country regional hospital for further investigations and expert management. His past medical history was unremarkable and he denied any history of tobacco or intravenous drug use (IVDU), chest trauma, sexually transmitted infection (STI) or open tuberculosis (TB) contact. He was diagnosed with pulmonary TB based on constitutional symptoms and chest X-ray findings, and had completed a 6-month course of anti-TB medications (isoniazid, rifampicin, pyrazinamide, and ethambutol) 7 weeks prior his visit to our institution. He was somewhat symptom-free for about 3 weeks when he developed recurrent episodes of breathlessness, awareness of heartbeats and coughing blood, which had gradually worsened and persisted for about 4 weeks prior this index visit.

On examination, he was a sick-looking but oriented and well-kempt young man. He had a blood pressure of 92/57 mmHg, pulse rate of 121 beats/minute, respiratory rate of 19 breaths/minute and temperature of 36.7 °C. His body mass index (BMI) was 21.2 kg/m^2^ (weight 59 kg and height 1.67 m). A physical examination revealed conjuctival and palmar pallor but there were no stigmata of connective tissue disorders, systemic vasculitides or congenital heart disease. A respiratory system examination revealed bilateral symmetrical chest movements; however, dullness and reduced breath sounds were noted on the mammary and inframammary regions bilaterally on percussion and auscultation respectively. An examination of the cardiovascular system revealed accentuated second heart sound (S_2_) with early diastolic (grade 1/6) and holosystolic (grade 2/6) murmurs at the pulmonic and tricuspid areas respectively.

**Fig. 1:**
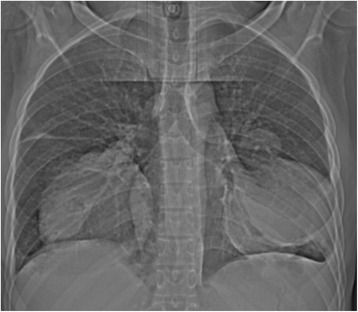
Chest X-ray (posteroanterior view) displaying bilateral, ovoid-shaped perihilar opacities extending to lower lung zones with loss of silhouette sign on the left cardiac border

**Fig. 2:**
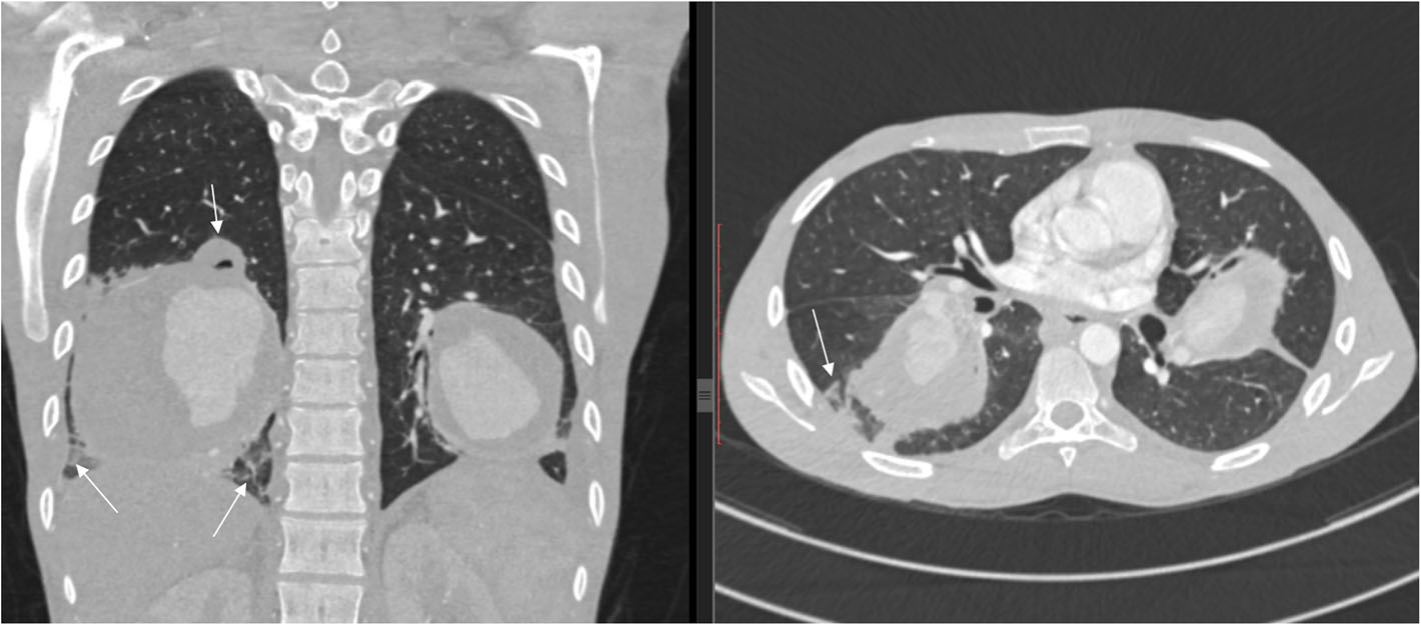
Computed tomography chest scan (coronal and axial views) showing bilateral pulmonary artery pseudoaneurysms with adjacent cystic changes (*arrows*), consolidations and tree-in-bud appearance

**Fig. 3:**
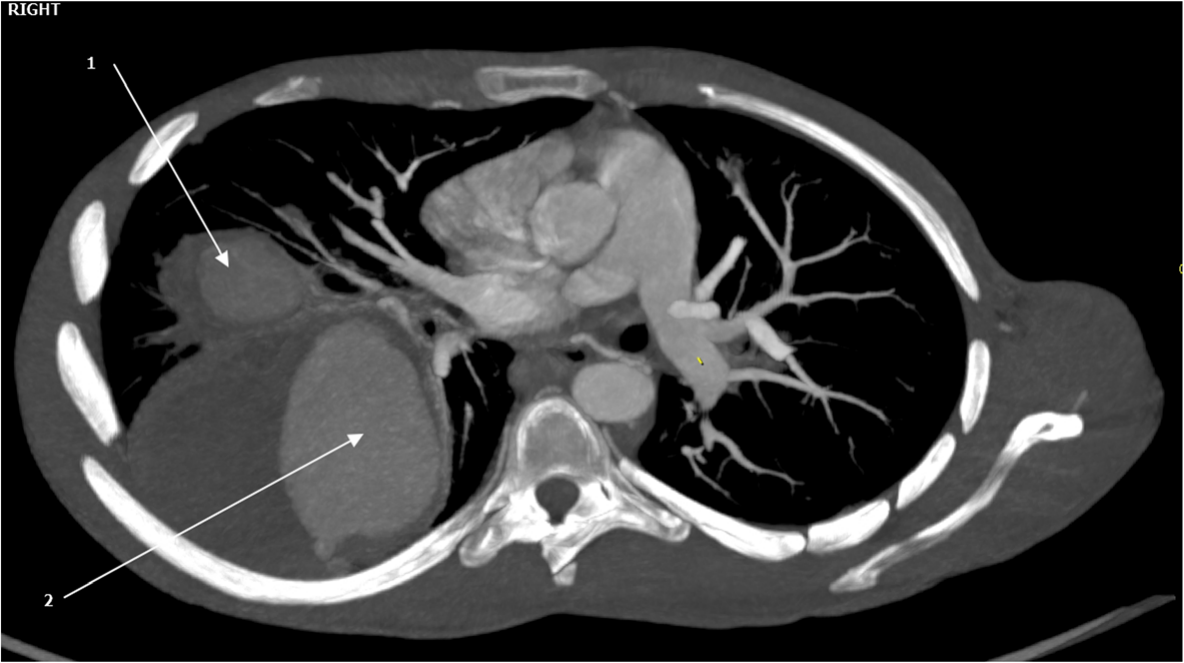
Computed tomography chest scan (axial and coronal views) showing multiple bilateral pulmonary artery pseudoaneurysms (*arrows*) with surrounding hematoma

**Fig. 4:**
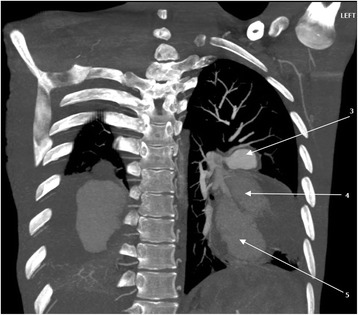
Computed tomography chest scan (axial and coronal views) showing multiple bilateral pulmonary artery pseudoaneurysms (*arrows*) with surrounding hematoma

**Fig. 5:**
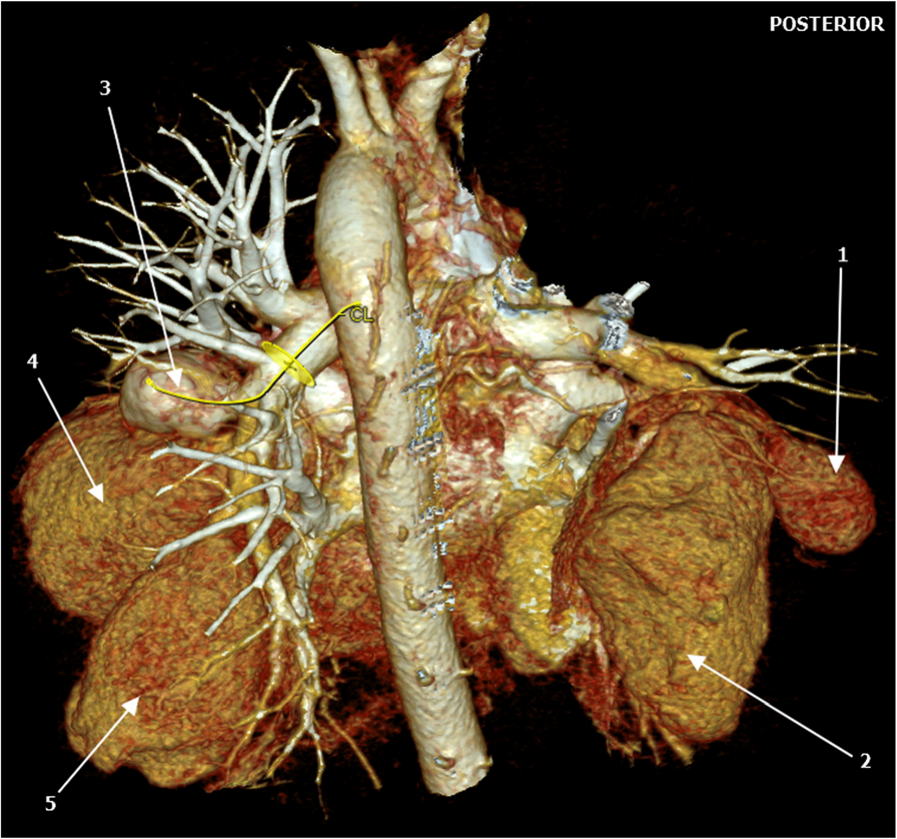
Volume-rendered computed tomography image showing multiple bilateral pulmonary pseudoaneurysms (*arrows*)

Hematological and biochemical tests revealed iron deficiency anemia [hemoglobin (Hb) 8.18 g/dL, mean corpuscular volume (MCV) 61.8 fL, mean corpuscular hemoglobin (MCH) 19.2 pg/cell and red cell distribution width (RDW) 21.9%], prolonged bleeding time [prothrombin time (PT) 14.6 s and partial thromboplastin time (PTT) 32.7 s], and mild hyponatremia [sodium concentration (Na^+^) 132 mmol/L]. Serological tests for human immunodeficiency virus (HIV), hepatitis B and C, and syphilis were all negative. Moreover, occult blood test was negative and an abdominal ultrasonography scan was unremarkable. A sputum culture revealed *Pseudomonas aeruginosa* 3++ sensitive to ciprofloxacin and gentamicin. A chest X-ray showed bilateral, ovoid-shaped perihilar opacities extending to lower lung zones with loss of silhouette sign on the left cardiac border (Fig. 1). An electrocardiogram (ECG) displayed a sinus tachycardia while echocardiography (ECHO) was evident for right ventricular hypertrophy, mild tricuspid regurgitation with estimated right ventricular systolic pressure (RVSP) of 29 mmHg (that is, mild pulmonary hypertension) and multiple focal dilatations along the right and left pulmonary arteries. Systolic functions were, however, preserved (ejection fraction 53%). A GeneXpert test was positive. A computed tomography (CT) scan of his chest showed bilateral multiple pulmonary artery pseudoaneurysms with surrounding hematoma together with adjacent cystic changes, consolidations and tree-in-bud appearance (Figs. 2, 3, 4, and 5). The aneurysms on the right pulmonary artery measured 3.81×2.55 cm and 6.96×5.34 cm whereas those on the left measured 2.61×2.03 cm, 6.85×4.45 cm and 7.05×4.03 cm respectively. His main pulmonary artery (2.55 cm), ascending (2.28 cm), arch (1.89 cm), and descending aorta (2.00 cm) had normal caliber and dimensions. We entertained a diagnosis of bilateral multiple pulmonary pseudoaneurysms associated with cavitary pulmonary tuberculosis and admitted him to the general medical ward. Intravenous ciprofloxacin 500 mg 12 hourly, bisoprolol 5 mg once daily, tadalafil (40 mg) and ferrous sulfate (270 mg) plus folic acid (300 mcg) were initiated. Moreover, our patient was counseled regarding surgery and prognosis of his condition; however, he refused to undergo any surgical procedure. After 9 days of hospitalization, our patient died of aneurismal rupture. Due to religious beliefs, relatives of the deceased refused an autopsy and one was not done.

## Discussion

Pulmonary artery aneurysms constitute <1% of aneurysms occurring in the thoracic cavity [29]. The cause could be idiopathic but many medical conditions including congenital heart defects, connective tissue disorders, systemic vasculitides, and infections are often associated with PAA [3, 4, 8, 9, 14, 16–24]. Clinical presentation is invariably nonspecific and roughly depends on the underlying etiology, location, and size of the aneurysm [9]. Owing to its largely vague presentation [4–7], noninvasive imaging techniques are crucial in reaching the diagnosis [8–13]; however, pulmonary angiography remains the gold standard diagnostic modality [2, 8, 19, 21, 23, 30–32]. About 20–60% of patients with PAA will die from aneurysm rupture while other serious complications including airway compression and intravascular thrombosis are not uncommon [9, 33–38].

Hemoptysis in a tuberculosis (TB) setting is relatively common, usually self-limiting or controlled by anti-TB drugs. Nevertheless, presence of a massive or several episodes of minor hemoptysis in TB is life-threatening, likely originating from the arterial system, and require early and aggressive intervention [39, 40]. Rasmussen’s aneurysm is a rare sequalae of pulmonary TB resulting from gradual weakening of the pulmonary artery wall from the adjacent tubercular cavity leading to thinning and pseudoaneurysm formation [36–39]. It has been reported in approximately 5% of autopsy series involving patients with chronic cavitary tuberculosis [39–44]. Despite absence of clear guidelines for treatment of PAA, several surgical techniques including aneurysmorrhaphy, Dacron graft replacement, pulmonary allograft replacement, and replacement with combination of Dacron prosthesis and stentless bioprosthesis are advocated [9, 19, 21, 28, 45, 46]. Furthermore, patients presenting with massive hemoptysis are usually treated with a bronchial artery embolization procedure [47]. However, such an embolization procedure technique requires special care especially in pseudoaneurysms as they are easily prone to rupture with resultant fatal bleeding [39, 43, 47].

Our patient refused to undergo any surgical procedure and consequently died of aneurismal rupture on the ninth day of hospitalization. Arguably, even if he had opted for surgery, the presence of multiple bilateral large pseudoaneurysms would have potentiated a high risk of fatal bleeding, among other complications, using any surgical technique. Nevertheless, watchful waiting, as in this case, portends a very poor prognosis. To the best of our knowledge, this was the first ever case of TB-associated PAA to be diagnosed and documented in Tanzania. We hope that upon its publication this case will sensitize practitioners to consider and assess for PAA whenever faced by hemoptysis not responding to otherwise routine therapy.

## Conclusions

In conclusion, presence of intractable hemoptysis among TB patients even after completion of an anti-TB course should raise an index of suspicion for PAA. Furthermore, despite its rarity, early recognition and timely surgical intervention is crucial in reducing morbidity and preventing the attributed mortality.

## Abbreviations

CT: Computed tomography
ECG: Electrocardiogram
ECHO: Echocardiography
Hb: Hemoglobin
IVDU: Intravenous drug use
MCH: Mean corpuscular hemoglobin
MCV: Mean corpuscular volume
MRI: Magnetic resonance imaging
Na^+^: Sodium concentration
PAA: Pulmonary artery aneurysm
PT: Prothrombin time
PTT: Partial thromboplastin time
RDW: Red cell distribution width
RVSP: Right ventricular systolic pressure
STI: Sexually transmitted infections
TB: Tuberculosis
